# Supplementation with dimethylglycine sodium salt improves lipid metabolism disorder in intrauterine growth-retarded pigs

**DOI:** 10.1016/j.aninu.2024.05.002

**Published:** 2024-06-01

**Authors:** Kaiwen Bai, Luyi Jiang, Tian Wang

**Affiliations:** a School of Biological and Chemical Engineering, Zhejiang University of Science and Technology, Hangzhou 310023, China; b College of Animal Science and Technology, Nanjing Agricultural University, Nanjing 210095, China; c College of Biology and Environmental Engineering, Zhejiang Shuren University, Hangzhou 310023, China; d Institute of Dairy Science, Ministry of Education Key Laboratory of Molecular Animal Nutrition, College of Animal Sciences, Zhejiang University, Hangzhou 310023, China

**Keywords:** Intrauterine growth retardation, Pig, Lipid metabolism, Mitochondrial function, Dimethylglycine sodium salt

## Abstract

This study aims to elucidate the mechanism of lipid metabolism disorder in intrauterine growth retardation (IUGR) pigs and the potential alleviating effects of dimethylglycine sodium salt (DMG-Na). A total of 60 male newborn piglets were selected for this study. Within each litter, one normal birth weight (NBW) male piglet (1.53 ± 0.04 kg) and two IUGR male piglets (0.76 ± 0.06 kg) were chosen based on their birth weight. The piglets were divided into three groups for the study: NBW pigs received a PBS gavage and a common basal diet (NBW-C group), IUGR pigs received the same PBS gavage and common basal diet (IUGR-C group), and IUGR pigs received a 70-mg DMG-Na gavage along with a common basal diet supplemented with 0.1% DMG-Na (IUGR-D group). At 150 d of age, all piglets underwent euthanasia by exsanguination following electrical stunning, after which plasma, liver, and longissimus dorsi (LM) samples were promptly collected. The IUGR-D group demonstrated improvements in plasma parameters (*P* < 0.05), with lower triglyceride and free fatty acid (FFA) values, and hormone levels (*P* < 0.05), with lower growth hormone, insulin, and homeostasis model assessment of insulin resistance values. Restoration of lipid metabolism was observed (*P* < 0.05), with lower triglyceride and FFA, and higher hepatic lipase and total lipase values in the liver, and lower triglyceride and FFA values in the LM. Mitochondrial ETC complexes showed increased levels (*P* < 0.05), including higher complex III values in the liver, and higher complex I, complex III, and complex V values in the LM. Enhanced levels of energy metabolites were noted (*P* < 0.05), with higher NAD^+^, NAD^+^/NADH, adenosine triphosphate, and mtDNA values, and lower NADH values in the liver and LM. Additionally, meat quality parameters showed improvement (*P* < 0.05), with higher pH 24 h and a∗ values, and lower drip loss 48 h, L∗, and b∗ values. The expressions of lipid metabolism and mitochondrial function-related genes and proteins were upregulated (*P* < 0.05) compared to the IUGR-C group. In conclusion, it was indicated that IUGR pigs experienced lipid metabolism disorders and diminished performance. However, supplementation with DMG-Na showed promise in mitigating these adverse physiological effects by safeguarding body tissues and modulating energy metabolism.

## 1 Introduction

Intrauterine growth retardation (IUGR) describes the delayed growth and development of fetuses in the maternal uterus (Sutherland and Black, 2023; Zhen and Li, 2023). This condition contributes to increased perinatal mortality, compromised postnatal growth performance, suboptimal feed utilization, and lipid metabolism disorder in IUGR piglets, ultimately resulting in substantial economic losses for livestock industry (Cheng et al., 2021). Disorders in lipid metabolism can lead to the accumulation or irregular distribution of lipids in IUGR piglets, which can adversely affect their growth and development (Cheng et al., 2020, 2021). Preliminary research indicates that IUGR newborn, suckling, and weaned piglets exhibit inferior growth performance, lipid metabolism, and functionality of the liver, small intestine, and skeletal muscle compared to their non-IUGR counterparts (Bai et al., 2021, 2022a,b,c,d; Cheng et al., 2020). Thus, through scientific feed additive preparation, proper feeding management, and suitable exercise training, growth performance, lipid metabolism, and meat quality in pigs can be enhanced.

The liver plays a crucial role in nutrition metabolism, contributing to the energy and material balance within the body (Lotto et al., 2023). Skeletal muscles provide structural support for the skeletal system and facilitate a wide range of movements and activities (Koves et al., 2023). It also serves as a major source of protein in pigs, crucial for their growth and development (Bishop and Hawley, 2022). The liver and skeletal muscle play vital roles in regulating lipid metabolism and are highly active in fatty acid oxidation, making lipid metabolism homeostasis crucial for the growth, development, and health of pigs (Imai et al., 2022; Druzak et al., 2023). These tissues are rich in mitochondria, the primary sites for fatty acid β-oxidation, which generates adenosine triphosphate (ATP) and heat energy via oxidative phosphorylation. However, mitochondrial oxidative metabolism inevitably produces reactive oxygen species (ROS) as by-products. Under normal conditions, ROS levels are balanced by intracellular antioxidants and contribute to signal transduction and metabolic processes. Yet, mitochondrial dysfunction can disrupt fatty acid oxidation, leading to lipid metabolism disorder and adverse effects on growth, development, and metabolic health. Notably, IUGR pigs often exhibit early-onset mitochondrial dysfunction, characterized by increased ROS production, impaired biosynthesis, oxidative phosphorylation, and reduced ATP production, persisting into adulthood (Bai et al., 2021, 2022a,b,c,d). Studies indicate that lipid metabolism disorders and ectopic fat deposition significantly contributes to the negative effects of IUGR (Chen et al., 2022; Zhang et al., 2023). Consequently, improving mitochondrial function in the liver and skeletal muscle of IUGR pigs has emerged as a crucial strategy to alleviate lipid metabolism disorders and mitigate associated complications.

Dimethylglycine sodium salt (DMG-Na) serves as a valuable feed additive in the pig industry, primarily enhancing the taste and nutritional value of pig feed. Its incorporation improves feed palatability, stimulates pigs' appetites, and subsequently enhances food intake, leading to improved growth rates and overall production performance. Additionally, DMG-Na acts as a nutritional supplement, providing essential amino acids that facilitate muscle growth and development in pigs. Previous research has demonstrated that dietary supplementation with DMG-Na effectively enhances growth performance, redox status, immune function, and the functionality of the liver, small intestine, and skeletal muscle in IUGR newborn, suckling, and weaned piglets (Bai et al., 2021, 2022a,b,c,d). However, there is limited research on the mechanism by which dietary DMG-Na, when added to a standard basal diet, alleviates IUGR-induced lipid metabolism disorders in pigs. Based on our series of studies investigating lipid metabolism in IUGR, we have identified several potential mechanisms by which DMG-Na may exert its effects. Firstly, DMG-Na is proposed to enhance liver and muscle function, which plays a crucial role in lipid metabolism. By improving liver and muscle function, DMG-Na may indirectly influence lipid metabolism processes. Secondly, DMG-Na possesses antioxidant properties, which could counteract oxidative stress known to impact lipid peroxidation and subsequently affect lipid metabolism. Moreover, DMG-Na enhances energy metabolism, and since lipid metabolism is closely intertwined with energy production and utilization, improvements in energy metabolism may indirectly regulate lipid metabolism (Bai et al., 2021, 2022a,b,c,d; Cheng et al., 2020, 2021). In the present study, we observed that the lipid metabolism disorder in the liver and longissimus dorsi muscle (LM) of IUGR pigs. Our objective was to investigate the beneficial effects of DMG-Na supplementation on lipid metabolism disorders in the liver and LM of IUGR pigs.

## 2 Material and methods

### 2.1 Animal ethics statement

All animal procedures adhered to the Laboratory Animals-Standards of Welfare and Ethics (DB32/T 2911-2016) issued by the Jiangsu Market Supervision and Administration Bureau. Additionally, all experimental protocols for animal care were approved by the Nanjing Agricultural University Institutional Animal Care and Use Committee (SYXK-2017-0027).

### 2.2 Animal experiment and sampling

During gestation, 100 healthy pregnant multiparous sows (Landrace × Yorkshire) were selected based on similar expected farrowing dates (within 3 d) and parity (second or third). These sows were fertilized by Duroc boars and fed a consistent gestating diet meeting the nutrient requirements outlined by the National Research Council (NRC, 2012). Following farrowing, 20 sows with litters of 11 to 13 live-born piglets meeting the criteria for IUGR were identified. A total of 60 male newborn piglets were then selected for the study due to the reliability and applicability of findings obtained from research involving male animals. Within each litter, 1 normal birth weight (NBW) male piglet (1.53 ± 0.04 kg) and 2 IUGR male piglets (0.76 ± 0.06 kg, mean ± SE) were chosen based on birth weight, following a previously established protocol (Wang et al., 2005). The pigs were subsequently divided into three groups based on their condition: NBW pigs, which received a PBS gavage from d 7 to 21 and were fed a standard basal diet from d 22 to 150 (NBW-C group); IUGR pigs, which underwent the same PBS gavage regimen from d 7 to 21 and were fed a standard basal diet from d 22 to 150 (IUGR-C group); IUGR pigs, which received a 70 mg DMG-Na gavage from d 7 to 21 and were fed a standard basal diet supplemented with 0.1% DMG-Na from d 22 to 150 (IUGR-D group). The dosage of DMG-Na was determined as optimal based on the results of our preliminary experiments (Bai et al., 2021, 2022a,b,c,d). Each group contained 20 pigs, randomly divided into 4 replicates, with each replicate housed individually in a separate pen. After birth, NBW and IUGR piglets were nursed interactively and allowed to suckle naturally. From 7 to 21 d of age, based on previously obtained daily milk collection data, 70 mg DMG-Na was dissolved in 2 mL PBS daily and administered to 20 IUGR-D piglets via gavage. The remaining 20 NBW-C and 20 IUGR-C piglets received 2 mL of PBS solution via gavage. Prior to the commencement of the trial, dedicated staff members visited each sow daily, providing gentle interaction to acclimate them to human presence and minimize stress responses. Throughout the weanling period, piglets were allowed ad libitum access to feed, carefully monitored by experienced workers from d 7 to 21 to ensure satiety. No stress-related behaviors were observed during the experiment, and piglets did not engage in foraging post-feeding. Housed in environmentally controlled plastic enclosures (1.5 m × 0.7 m × 0.7 m) maintained at an ambient temperature of 33 °C, the piglets had unrestricted access to water. Following weaning at 21 d of age, NBW-C, IUGR-C, and IUGR-D piglets were fed a basal diet or basal diet supplemented with 0.1% DMG-Na until 150 d of age. Individually housed in plastic-floored pens (1 m × 0.6 m), the piglets were accommodated in an environmentally controlled room maintained at a temperature of 28 °C. Access to feed and water was provided ad libitum for the duration of the study.

DMG-Na (99.9% of purity, obtained from Qilu Sheng Hua Pharmaceutical Co., Ltd., Shandong, China) was stored in a −20 °C refrigerator. Previous studies conducted by our team have established the safety of DMG-Na as an antioxidant substance, with no reported side effects (Bai et al., 2021, 2022a, b, c, d). The composition and nutrient content of the basal diet for both sows and pigs meet the National Research Council (NRC, 2012) nutrient requirements and can be found in Tables S1 and Table 1, respectively. All chemical analyses of the feed samples were measured in triplicate before the raising experiments. Samples were analyzed for crude protein (CP) by combustion (method 999.03; AOAC, 2007) using a Rapid N cube apparatus (Elementar Americas Inc., Mt. Laurel, NJ, USA), for acid ether extract (AEE) by acid hydrolysis with 3 mol/L HCl (Sanderson, 1986), for crude fiber (CF) following Holst (1973), for amino acids (AA) with a Hitachi L8800 Amino Acid Analyzer (Hitachi High Technologies America Inc., Pleasanton, CA) using ninhydrin for postcolumn derivatization and norleucine as the internal standard, for calcium (Ca) and phosphorus (P) using inductively coupled plasma spectroscopy (method 985.01 A and B; AOAC, 2007). The gross energy (GE) of ingredients, diets, feces, and urine was measured using an Automatic Isoperibol Oxygen Bomb Calorimeter (Parr 1281 Calorimeter; Parr Instrument Co., Moline, IL, USA). The digestible energy (DE) and metabolizable energy (ME) contents of the diets were calculated using the following equations: DE = (GE_in_ − GE_out1_)/F_in_, ME = (GE_in_ − GE_out2_)/F_in_, where DE is the DE content in diets (MJ/kg), GE_in_ is the total GE intake (MJ), GE_out1_ is the GE content in feces (MJ), GE_out2_ is the GE content in feces and urine (MJ), F_in_ is the total feed intake (kg). At 150 d of age, all piglets underwent weighing after a 12 h feed deprivation period to assess body weight gain (BWG), feed intake (FI), and the ratio of BWG to FI (G:F) as indicators of feed conversion efficiency. Blood samples were then collected from the precaval vein, and plasma was separated by centrifugation at 3500 ×*g* for 15 min at 4 °C. The plasma was subsequently stored at −80 °C for subsequent analysis. Following blood collection, the pigs were euthanized via exsanguination after electrical stunning, and liver as well as LM samples were promptly collected. A portion of liver and LM samples from the left side were preserved for histological morphology studies, while the remaining portions (approximately 30 g) were rapidly frozen in liquid nitrogen and stored at −80 °C for further analysis.

**Table 1:** Composition and nutrient level of the diets for experimental pigs (as-fed basis, %).^1^

Item	Starter diet	Grower diet	Finisher diet
Ingredients			
Corn	63.4	60.22	60.1
Full-fat rice bran	0	7.5	8
Flour	5	0	0
Wheat middlings	0	10	10
Bran	0	2	6
Soybean meal	17.5	16.5	0
Fermented soybean meal	2.5	0	0
De-hulled soybean meal	0	0	13.1
Soybean oil	2.3	0	0
Fish meal	1.25	0	0
Soybean protein concentrate	2.5	0	0
Glucose	1.25	0	0
Phospholipid powder	1	0	0
Limestone	0.91	1.4	1
CaHPO_4_	0.46	0.49	0
Salt	0.41	0.35	0.38
Lysine	0.42	0.43	0.35
Methionine	0.1	0.11	0.07
Premix^2^^,^^3^^,^^4^	1	1	1
Total	100	100	100
Analyzed nutrient level			
CP	18.3	15.6	14.6
AEE	3	3.82	4.15
CF	6		
Lysine	1.24	0.98	0.91
Ca	0.78	0.7	0.59
P	0.38	0.57	0.51
Calculated nutrient level			
DE, MJ/kg	14.42	12.6	12.43
ME, MJ/kg	13.71	11.98	11.82

ME = metabolizable energy; AEE = acid-hydrolyzed ether extract.

1 Starter diet, the diet for pigs weighing from 5 to 10 kg; Grower diet, the diet for pigs weighing from 10 to 30 kg; Finisher diet, the diet for pigs weighing from 30 to 100 kg.

2 Provided per kilogram of the starter diet: vitamin A, 15,000 IU; vitamin B_1_, 3 mg; vitamin B_2_, 6 mg; vitamin B_6_, 7 mg; vitamin B_12_, 0.03 mg; vitamin D_3_, 3200 IU; vitamin E, 22 mg; vitamin K_3_, 3 mg; niacin, 30 mg; pantothenic acid, 15 mg; folic acid, 1.2 mg; biotin, 0.08 mg; choline chloride, 500 mg; Fe, 120 mg; Cu, 120 mg; Zn, 110 mg; Mn, 43 mg; I, 0.7 mg; Se, 0.3 mg.

3 Provided per kilogram of the grower diet: vitamin A, 6400 IU; vitamin B_1_, 1.2 mg; vitamin B_2_, 4 mg; vitamin B_6_, 3 mg; vitamin B_12_, 0.04 mg; vitamin D_3_, 3200 IU; vitamin E, 16 mg; vitamin K_3_, 2 mg; Niacin, 30 mg; pantothenic acid, 15 mg; folic acid, 0.6 mg; biotin, 0.05 mg; choline chloride, 400 mg; Fe, 100 mg; Cu, 18 mg; Zn, 80 mg; Mn, 27 mg; I, 0.75 mg; Se, 0.3 mg.

4 Provided per kilogram of the finisher diet: vitamin A, 6500 IU; vitamin B_1_, 2 mg; vitamin B_2_, 4 mg; vitamin B_6_, 2 mg; vitamin B_12_, 0.04 mg; vitamin D_3_, 2,200 IU; vitamin E, 25 mg; vitamin K_3_, 2 mg; niacin, 30 mg; pantothenic acid, 15 mg; folic acid, 0.6 mg; biotin, 0.05 mg; choline chloride, 300 mg; Fe, 60 mg; Cu, 21 mg; Zn, 60 mg; Mn, 15 mg; I, 0.75 mg; Se, 0.3 mg; Ca, 5.1 g; P, 0.6 g.

### 2.3 Histological morphology assays

**Fig. 1:**
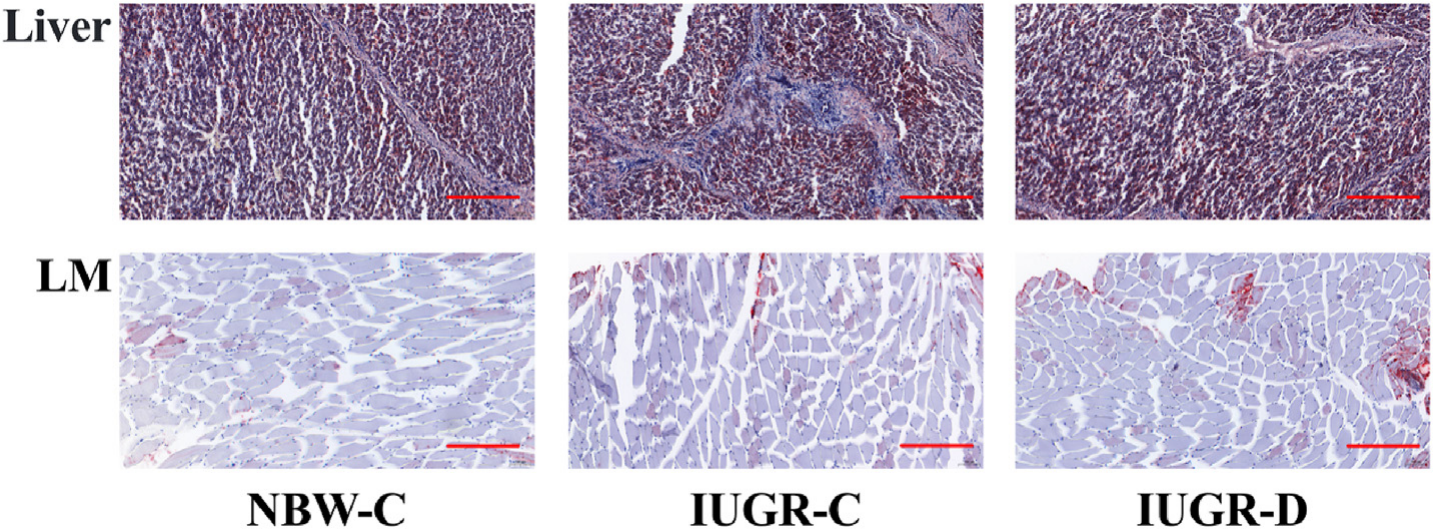
Supplementation with DMG-Na improves histological morphology of the liver and LM in IUGR pigs. (A) Oil red O staining of the liver; scale bars represent 100 μm. (B) Oil red O staining of the LM; scale bars represent 100 μm. DMG-Na = dimethylglycine sodium salt; LM = longissimus dorsi; NBW = normal birth weight; IUGR = intrauterine growth restriction. NBW-C = NBW pigs fed a standard basal diet; IUGR-C = IUGR pigs fed a standard basal diet; IUGR-D = IUGR pigs fed a standard basal diet supplemented with 0.1% DMG-Na.

Liver and LM samples from the pigs were processed into frozen sections, with each sample sliced into sections 5 μm thick. Oil Red O staining was conducted following standard protocols to visualize lipid droplets within the tissue sections (Fig. 1). Images of the stained sections were captured using an optical binocular microscope (Olympus BX5; Olympus Optical Co. Ltd, Tokyo, Japan) equipped with a digital camera (Nikon H550L; Nikon, Tokyo, Japan). This imaging setup allowed for the visualization and documentation of lipid accumulation within the liver and LM tissues.

### 2.4 Plasma parameter assays

The levels of glucose (A154-1-1, glucose oxidase method), total cholesterol (TC; A111-1-1, single-reagent GPO-PAP method), triglycerides (F001-1-1, TG enzyme method), free fatty acids (FFA; A042-1-1, colorimetry), high-density lipoprotein cholesterol (HDL-C; A112-2-1, two-reagent direct method), low-density lipoprotein cholesterol (LDL-C; A113-2-1, two-reagent direct method), creatinine (C011-2-1, sarcosine oxidase method), and urea nitrogen (UN; C013-2-1, urease method) in the plasma were quantified using commercially available kits, following the manufacturer's instructions (Nanjing Jiancheng Bioengineering Institute, Nanjing, China).

The levels of glucagon (GC; H183-1-2), growth hormone (GH; H091-1-2), and insulin-like growth factor I (IGF I; H041-1-2) in the plasma were quantified using corresponding enzyme-linked immunosorbent assay (ELISA) kits following the manufacturer's instructions (Nanjing Jiancheng Bioengineering Institute). Similarly, a commercial ELISA kit from Nanjing Jiancheng Bioengineering Institute was utilized to measure the plasma insulin (H203-1-2) level. The sensitivity limit for insulin determination was 1 μIU/mL, and both the inter- and intra-assay coefficients of variation were less than 15%. To calculate insulin resistance, the homeostasis model assessment of insulin resistance (HOMA-IR) was applied using the following formula:

$$\text{HOMA}-\text{IR}=\frac{\text{Fasting}\;\text{plasma}\;\text{insulin}\;\left(\mu \text{IU}/\text{mL}\right)\times \text{Fasting}\;\text{plasma}\;\text{glucose}\;\left(\text{mmol}/L\right)}{22.5}$$

### 2.5 Lipid metabolism of liver and LM assays

The liver and LM samples were homogenized in 0.9% sodium chloride solution on ice and centrifuged at 3500 × *g* for 15 min at 4 °C, respectively. The concentrations of TC, triglycerides, and FFA, as well as the activities of lipoprotein lipase (LPL, A067-1-2, colorimetry), hepatic lipase (HL, A067-1-2, colorimetry), and total lipase (TL, A067-1-2, colorimetry) in the liver, were determined using corresponding commercial kits obtained from Nanjing Jiancheng Bioengineering Institute, China following the manufacturer's instructions. Similarly, the levels of triglycerides and FFA in the LM were measured using the appropriate commercial kits from Nanjing Jiancheng Bioengineering Institute, China, following the manufacturer's instructions.

### 2.6 Mitochondrial ETC complexes of liver and LM assays

Hepatic and LM mitochondria were isolated utilizing the mitochondria isolation kit from Nanjing Jiancheng Bioengineering Institute, China. Subsequently, the activities of hepatic and LM mitochondrial electron transport chain complexes, including complexes I (A089-1-1, NADH-coenzyme Q reductase method), II (A089-2-1, succinate-coenzyme Q reductase method), III (A089-3-1, coenzyme Q-cytochrome c reductase method), IV (A089-4-1, orthocytochrome c oxidoreductase method), and V (A089-5-1, F0F1-ATPase/ATP synthase method), were assessed using commercial kits sourced from Nanjing Jiancheng Bioengineering Institute, China.

### 2.7 Energy metabolites of liver and LM assays

The concentrations of NAD^+^/NADH (A114-1-1, colorimetry) and ATP (A095-1-1, colorimetry) in both liver and LM samples were assessed using the corresponding assay kits obtained from Nanjing Jiancheng Institute of Bioengineering. To determine the NAD^+^/NADH concentrations, 50 μg of liver and LM mitochondrial samples were utilized. NAD^+^ and NADH were extracted from acidic and alkaline extracts. NADH was then utilized to reduce oxidized thiazole blue (MTT) to Formazan in the presence of phenazine methosulfate (PMS), and the resulting absorbance was measured at 570 nm. NAD^+^ was reduced to NADH by ethanol dehydrogenase and further detected via MTT reduction. For the assessment of ATP content, 30 μg of liver and LM mitochondrial samples were employed. Creatine kinase catalyzed the conversion of adenosine triphosphate and creatine to generate phosphocreatine, which was detected using the phosphomolybdate colorimetric assay. The absorbance was then measured at 636 nm. Additionally, glycogen content in the liver and LM mitochondrial samples was measured using 100 μg of each sample. Glycogen (A043-1-1, colorimetry) was dehydrated with concentrated sulfuric acid to produce glutaraldehyde derivatives, which formed blue compounds with anthracenes. The resulting colorimetric reaction was quantified using a standard glucose solution, and the absorbance was measured at 620 nm.

The mitochondrial DNA (mtDNA) copy number in both liver and LM samples was determined using an RT-qPCR kit (Tli RNaseH Plus; Takara Bio, Inc., Otsu, Japan). In brief, a 20 μL PCR mixture was prepared, comprising 10 μL SYBR Premix Ex Taq (2×), 0.4 μL upstream primer, 0.4 μL downstream primer, 0.4 μL ROX dye (50×), 6.8 μL ultra-pure water, and 2 μL cDNA template. For the mtDNA copy number determination, the upstream primer sequence for the Mt D-loop gene was 5′-AGGACTACGGCTTGAAAAGC-3′, and the downstream primer sequence was 5′-CATCTTGGCATCTTCAGTGCC-3′, resulting in a target fragment length of 198 bp. For normalization, β-actin was used as an internal standard. The upstream primer sequence for β-actin was 5′-TTCTTGGGTATGGAGTCCTG-3′, and the downstream primer sequence was 5′-TAGAAGCATTTGCGGTGG-3′, resulting in a target fragment length of 150 bp. The fold expression of each gene was calculated according to the 2^−ΔΔCt^ method, where the cycle threshold (Ct) values of the target gene were normalized to the Ct values of β-actin, and the relative expression levels were compared to a control sample.

### 2.8 Meat quality assays

At 150 d of age, LM meat samples were collected from individuals for the analysis of physicochemical properties. The pH values of the meat were determined using a digital pH meter (NWKbinar pH, K-21, Landberg, Germany). The drip loss of meat samples was measured by suspending them and standardizing for surface area in cups at 4 °C for 48 h. The initial and final weight of each sample was recorded to calculate drip loss. Meat samples were cooked to a final core temperature of 75 °C, and the values of cooking loss were measured by comparing the weights before and after cooking. After cooking, three cores of each sample (1.27 cm diameter) parallel to the longitudinal orientation of muscle fibers were used for shear force measurements, which serve as an indicator of meat tenderness. The meat color of samples was measured at 3 different locations across the meat using a Minolta colorimeter (CR-300, Tokyo, Japan), and expressed as lightness (L∗), redness (a∗), and yellowness (b∗).

### 2.9 Quantitative reverse transcription PCR assays

Quantitative reverse transcription PCR (qPCR) was conducted following established protocols in our laboratory (Mohamed et al., 2010). Briefly, total RNA was extracted from liver and LM samples using Trizol Reagent (TaKaRa, Dalian, China), and then reverse-transcribed using a commercial kit (Perfect Real Time, SYBR PrimeScript, TaKaRa) according to the manufacturer's instructions. The mRNA expression levels of specific genes were quantified via real-time PCR using SYBR Premix Ex Taq II (Tli RNaseH Plus) and an ABI 7300 Fast Real-Time PCR detection system (Applied Biosystems, Foster City, CA). The SYBR Green PCR reaction mixture comprised 10 μL SYBR Premix Ex Taq (2 ×), 0.4 μL of forward and reverse primers each, 0.4 μL of ROX reference dye (50 ×), 6.8 μL of ddH2O, and 2 μL of cDNA template. Each sample was amplified in triplicate. The fold expression of each gene was determined using the 2^-ΔΔCt^ method, with the β-actin gene serving as an internal standard. The primer sequences utilized are provided in Table S3. Total RNA was extracted from liver and LM samples using Trizol Reagent (TaKaRa, Dalian, China), and then reverse-transcribed using a commercial kit (Perfect Real Time, SYBR PrimeScript, TaKaRa) according to the manufacturer's instructions. The mRNA expression levels of specific genes were quantified via real-time PCR using SYBR® Premix Ex Taq II (Tli RNaseH Plus) and an ABI 7300 Fast Real-Time PCR detection system (Applied Biosystems, Foster City, CA). The SYBR Green PCR reaction mixture comprised 10 μL SYBR Premix Ex Taq (2×), 0.4 μL of forward and reverse primers each, 0.4 μL of ROX reference dye (50×), 6.8 μL of ddH_2_O, and 2 μL of cDNA template. Each sample was amplified in triplicate. The fold expression of each gene was determined using the 2^−ΔΔCt^ method, with the β-actin gene serving as an internal standard. The primer sequences utilized are provided in Table S2.

### 2.10 Western blotting assays

Total protein was extracted from liver and LM samples using radioimmunoprecipitation assay (RIPA) lysis buffer containing protease inhibitor cocktail (Beyotime Institute of Biotechnology, Jiangsu, China). Nuclear protein extraction from samples was carried out using a Nuclear Protein Extraction Kit (Beyotime Institute of Biotechnology). The concentrations of total cellular protein and nuclear protein in the samples were quantified using the bicinchoninic acid protein assay kit (Beyotime Institute of Biotechnology). Antibodies against the relevant proteins were purchased from Cell Signaling Technology (Danvers, MA, USA). Equal amounts of protein were separated by sodium dodecyl sulfate polyacrylamide gel electrophoresis (SDS-PAGE) gel electrophoresis and then transferred onto polyvinylidene difluoride membranes. The membranes were blocked with blocking buffer (5% bovine serum albumin in Tris-buffered saline containing 1% Tween 20) for 1 h at room temperature and then probed with primary antibodies (dilution: 1:1000) against peroxisome proliferator-activated receptor-γ (PPARγ; #2435), silent information regulator 1 (Sirt1; #9475S), PPARγ coactivator-1 α (PGC1α; #2178S), AMP-activated protein kinase (AMPK; #2532), and α-Tubulin (#2125S) overnight at 4 °C. After washing with Tris-buffered saline containing 0.05% Tween-20, the membranes were incubated with an appropriate secondary antibody for 1 h at room temperature. The blots were then visualized using enhanced chemiluminescence reagents (ECL-Kit, Beyotime, Jiangsu, China), and images were captured using the Luminescent Image Analyzer LAS-4000 system (Fujifilm Co.). Quantitative analysis of the bands was performed using ImageJ 1.42 q software (NIH, Bethesda, MD, USA).

### 2.11 Statistical analysis

The assumptions of equality of variances and normal distribution were assessed for all variables using the Shapiro–Wilk test and Q–Q graphics test. To identify differences between groups, a One-way nonparametric Kruskal–Wallis ANOVA with Dunnett's post-hoc test was utilized. All data were analyzed using Statistical Analysis System software (version 9.1; SAS Institute, Inc., Cary, NC, USA) and are presented as mean ± SE. Statistical significance was defined as *P* < 0.05. Each assay was conducted at least three times using a total of 60 pigs.

## 3 Results

### 3.1 Growth performance

All of the pigs remained healthy throughout the study period, and no mortality was observed in any of the groups. The growth performance results are presented in Table 2. Compared with the NBW-C group, the IUGR-C group exhibited significantly lower (*P* < 0.05) levels of initial body weight (IBW) and BWG. However, no differences were observed between the IUGR-D group and the IUGR-C group across the growth performance parameters.

**Table 2:** Supplementation with DMG-Na improves the growth performance of IUGR pigs.^1^

Item	NBW-C^2^	IUGR-C^3^	IUGR-D^4^
IBW, kg	1.52 ± 0.11^a^	0.75 ± 0.05^b^	0.75 ± 0.07^b^
FBW, kg	101.17 ± 8.03	84.26 ± 6.06	89.16 ± 7.55
BWG, kg	99.65 ± 6.07^a^	83.51 ± 5.44^b^	88.41 ± 5.18^b^
FI, kg	239.51 ± 18.18	224.86 ± 18.57	212.06 ± 19.86
G: F	0.42 ± 0.03	0.37 ± 0.03	0.42 ± 0.04

DMG-Na = dimethylglycine sodium salt; NBW = normal birth weight; IUGR = intrauterine growth restriction; IBW = initial body weight; FBW = final body weight; BWG = body weight gain; FI = feed intake; G:F = the ratio of body weight gain to feed intake.

^a, b^ Within a row, means with different superscripts indicated significantly different (*P* < 0.05).

1 Values are expressed as mean ± standard error.

2 NBW-C = NBW pigs fed a standard basal diet.

3 IUGR-C = IUGR pigs fed a standard basal diet.

4 IUGR-D = IUGR pigs fed a standard basal diet supplemented with 0.1% DMG-Na.

### 3.2 Plasma parameter assays

The results of plasma lipid metabolism parameters are depicted in Table 3. Compared to the NBW-C group, the IUGR-C group exhibited significantly higher (*P* < 0.05) levels of glucose and FFA, while showing lower (*P* < 0.05) levels of triglyceride and LDL-C. Conversely, the IUGR-D group demonstrated lower (*P* < 0.05) levels of FFA and higher (*P* < 0.05) levels of triglyceride compared to the IUGR-C group.

**Table 3:** Supplementation with DMG-Na improves the plasma parameters in IUGR pigs.^1^

Item	NBW-C^2^	IUGR-C^3^	IUGR-D^4^
Glucose, mmol/L	4.03 ± 0.29^b^	5.46 ± 0.44^a^	4.96 ± 0.41^a^
TC, mmol/L	4.61 ± 0.31	4.32 ± 0.28	4.43 ± 0.33
Triglyceride, mmol/L	0.48 ± 0.03^a^	0.20 ± 0.02^c^	0.35 ± 0.02^b^
FFA, μmol/L	198.33 ± 17.88^b^	283.11 ± 22.56^a^	209.64 ± 18.72^b^
HDL-C, mmol/L	2.01 ± 0.15	2.27 ± 0.17	2.07 ± 0.15
LDL-C, mmol/L	2.46 ± 0.18^a^	1.75 ± 0.14^b^	1.93 ± 0.16^b^
Creatinine, μmol/L	132.77 ± 12.55	165.29 ± 15.43	140.56 ± 13.19
UN, mmol/L	5.22 ± 0.4	6.48 ± 0.52	5.79 ± 0.5

DMG-Na = dimethylglycine sodium salt; NBW = normal birth weight; IUGR = intrauterine growth restriction; TC = total cholesterol; FFA = free fatty acids; HDL-C = high-density lipoprotein cholesterol; LDL-C = low-density lipoprotein cholesterol; UN = urea nitrogen.

^a, b, c^ Within a row, means with different superscripts indicated significantly different (*P* < 0.05).

1 Values are expressed as mean ± standard error (*n* = 20 pigs).

2 NBW-C = NBW pigs fed a standard basal diet.

3 IUGR-C = IUGR pigs fed a standard basal diet.

4 IUGR-D = IUGR pigs fed a standard basal diet supplemented with 0.1% DMG-Na.

The results of plasma hormone concentrations are illustrated in Table 4. In comparison to the NBW-C group, the IUGR-C group displayed higher (*P* < 0.05) levels of GH, IGF I, insulin, and HOMA-IR. Conversely, the IUGR-D group showed lower (*P* < 0.05) levels of GH, insulin, and HOMA-IR compared to the IUGR-C group.

**Table 4:** Supplementation with DMG-Na improves the plasma hormone parameter in IUGR pigs.^1^

Item	NBW-C^2^	IUGR-C^3^	IUGR-D^4^
GC, pg/mL	366.75 ± 33.79	288.72 ± 25.46	326.28 ± 30.27
GH, ng/mL	13.01 ± 1.17^b^	21.06 ± 2.02^a^	15.28 ± 1.11^b^
IGF I, ng/mL	9.54 ± 0.8^b^	13.15 ± 1.17^a^	11.77 ± 1.05^ab^
Insulin, μIU/mL	10.15 ± 0.91^b^	32.05 ± 2.75^a^	12.33 ± 1.13^b^
HOMA-IR index	1.36 ± 0.10^b^	8.26 ± 0.65^a^	2.07 ± 0.13^b^

DMG-Na = dimethylglycine sodium salt; NBW = normal birth weight; IUGR = intrauterine growth restriction; GC = glucagon; GH = growth hormone; IGF I = insulin-like growth factor I; HOMA-IR = homeostasis model assessment-insulin resistance.

^a, b^ Within a row, means with different superscripts indicated significantly different (*P* < 0.05).

1 Values are expressed as mean ± standard error (*n* = 20 pigs).

2 NBW-C = NBW pigs fed a standard basal diet.

3 IUGR-C = IUGR pigs fed a standard basal diet.

4 IUGR-D = IUGR pigs fed a standard basal diet supplemented with 0.1% DMG-Na.

### 3.3 Lipid metabolism of liver and LM assays

The results of hepatic lipid metabolism parameters are illustrated in Table 5. In comparison to the NBW-C group, the IUGR-C group displayed significantly higher (*P* < 0.05) levels of TC, triglyceride, and FFA, while exhibiting lower (*P* < 0.05) levels of LPL, HL, and TL. Conversely, the IUGR-D group showed lower (*P* < 0.05) levels of triglyceride and FFA, and higher (*P* < 0.05) levels of LPL, HL, and TL compared to the IUGR-C group.

**Table 5:** Supplementation with DMG-Na improves the lipid metabolism of liver and LM in IUGR pigs.^1^

Item	NBW-C^2^	IUGR-C^3^	IUGR-D^4^
Liver			
TC, μmol/g protein	111.82 ± 10.28^b^	144.39 ± 13.34^a^	135.28 ± 12.19^a^
Triglyceride, μmol/g protein	133.72 ± 12.34^b^	223.56 ± 20.55^a^	163.15 ± 14.96^b^
FFA, μmol/g protein	0.92 ± 0.07^c^	2.25 ± 0.15^a^	1.54 ± 0.13^b^
LPL, U/mg protein	1.05 ± 0.07^a^	0.61 ± 0.04^b^	0.82 ± 0.06^ab^
HL, U/mg protein	1.20 ± 0.08^a^	0.36 ± 0.02^b^	0.95 ± 0.06^a^
TL, U/mg protein	2.12 ± 0.17^a^	1.00 ± 0.08^b^	1.95 ± 0.18^a^
LM			
Triglyceride, μmol/g protein	151.95 ± 13.38^b^	243.55 ± 22.53^a^	182.38 ± 18.02^b^
FFA, μmol/g protein	5.66 ± 0.42^b^	7.42 ± 0.66^a^	6.17 ± 0.58^b^

DMG-Na = dimethylglycine sodium salt; LM = longissimus dorsi; NBW = normal birth weight; IUGR = intrauterine growth restriction; TC = total cholesterol; FFA = free fatty acids; LPL = lipoprotein lipase; HL = hepatic lipase; TL = total lipase.

^a, b, c^ Within a row, means with different superscripts indicated significantly different (*P* < 0.05).

1 Values are expressed as mean ± standard error (*n* = 20 pigs).

2 NBW-C = NBW pigs fed a standard basal diet.

3 IUGR-C = IUGR pigs fed a standard basal diet.

4 IUGR-D = IUGR pigs fed a standard basal diet supplemented with 0.1% DMG-Na.

The results of LM lipid metabolism parameters are presented in Table 5. Compared to the NBW-C group, the IUGR-C group demonstrated significantly higher (*P* < 0.05) levels of triglyceride and FFA. However, the IUGR-D group exhibited lower (*P* < 0.05) levels of triglyceride and FFA compared to the IUGR-C group.

### 3.4 Mitochondrial ETC complexes of liver and LM assays

The results of hepatic mitochondrial ETC complexes are depicted in Table 6. In comparison to the NBW-C group, the IUGR-C group exhibited significantly lower (*P* < 0.05) levels of complexes II and III. Conversely, the IUGR-D group showed higher (*P* < 0.05) levels of complex III compared to the IUGR-C group.

**Table 6:** Supplementation with DMG-Na improves the mitochondrial ETC complexes of liver and LM in IUGR pigs.^1^

Item	NBW-C^2^	IUGR-C^3^	IUGR-D^4^
Liver, nmol/min per mg protein			
Complex I	511.32 ± 50	666.75 ± 63.85	606.78 ± 58.86
Complex II	13.06 ± 1.22^a^	6.61 ± 0.61^b^	10.11 ± 0.97^ab^
Complex III	155.68 ± 13.29^a^	91.34 ± 8.86^b^	133.29 ± 13.2^a^
Complex IV	4.12 ± 0.38	3.29 ± 0.30	3.88 ± 0.36
Complex V	13.81 ± 1.20	15.22 ± 1.44	14.75 ± 1.42
LM, nmol/min per mg protein			
Complex I	761.59 ± 75.22^a^	236.61 ± 22.91^b^	594.93 ± 58.85^a^
Complex II	5.88 ± 0.55^a^	3.12 ± 0.30^b^	4.55 ± 0.44^ab^
Complex III	76.24 ± 7.54^a^	26.77 ± 2.66^c^	53.86 ± 5.15^b^
Complex IV	61.26 ± 5.99	50.78 ± 4.65	55.43 ± 5.16
Complex V	18.22 ± 1.76^a^	6.11 ± 0.64^b^	15.84 ± 1.26^a^

DMG-Na = dimethylglycine sodium salt; LM = longissimus dorsi; NBW = normal birth weight; IUGR = intrauterine growth restriction.

^a, b, c^ Within a row, means with different superscripts indicated significantly different (*P* < 0.05).

1 Values are expressed as mean ± standard error (*n* = 20 pigs).

2 NBW-C = NBW pigs fed a standard basal diet.

3 IUGR-C = IUGR pigs fed a standard basal diet.

4 IUGR-D = IUGR pigs fed a standard basal diet supplemented with 0.1% DMG-Na.

The results of LM mitochondrial ETC complexes are presented in Table 6. Compared to the NBW-C group, the IUGR-C group demonstrated significantly lower (*P* < 0.05) levels of complexes I, II, III, and V. However, the IUGR-D group exhibited higher (*P* < 0.05) levels of complexes I, III, and V compared to the IUGR-C group.

### 3.5 Energy metabolites of liver and LM assays

The results of hepatic energy metabolites are illustrated in Table 7. In comparison to the NBW-C group, the IUGR-C group exhibited a significantly higher (*P* < 0.05) level of NADH and significantly lower (*P* < 0.05) levels of NAD^+^, NAD^+^/NADH ratio, ATP, and mtDNA. Conversely, the IUGR-D group showed lower (*P* < 0.05) NADH levels and higher (*P* < 0.05) levels of NAD^+^, NAD^+^/NADH ratio, ATP, and mtDNA compared to the IUGR-C group.

**Table 7:** Supplementation with DMG-Na improves the energy metabolites of liver and LM in IUGR pigs.^1^

Item	NBW-C^2^	IUGR-C^3^	IUGR-D^4^
Liver			
NAD^+^, μmol/g	5.44 ± 0.41^a^	2.52 ± 0.22^b^	4.16 ± 0.4^a^
NADH, μmol/g	1.35 ± 0.11^b^	2.89 ± 0.25^a^	1.73 ± 0.16^b^
NAD^+^/NADH	4.03 ± 0.36^a^	0.87 ± 0.07^c^	2.40 ± 0.22^b^
ATP, nmol/g	62.36 ± 5.9^a^	30.22 ± 2.87^b^	54.56 ± 5.12^a^
mtDNA, % of NBW-C	1.00 ± 0.05^a^	0.48 ± 0.03^b^	0.88 ± 0.06^a^
LM			
NAD^+^, μmol/g	4.81 ± 0.36^a^	3.29 ± 0.30^c^	4.09 ± 0.38^a^
NADH, μmol/g	1.44 ± 0.12^b^	2.96 ± 0.16^a^	2.03 ± 0.17^b^
NAD^+^/NADH	3.34 ± 0.32^a^	1.11 ± 0.11^c^	2.01 ± 0.20^b^
ATP, nmol/g	162.68 ± 15.47^a^	73.55 ± 6.94^b^	103.98 ± 9.92^b^
mtDNA, % of NBW-C	1.00 ± 0.07^a^	0.49 ± 0.03^b^	0.86 ± 0.08^a^

DMG-Na = dimethylglycine sodium salt; LM = longissimus dorsi; NBW = normal birth weight; IUGR = intrauterine growth restriction; NADH = nicotinamide adenine dinucleotide; ATP = adenosine triphosphate; mtDNA = mitochondrial DNA.

^a, b, c^ Within a row, means with different superscripts indicated significantly different (*P* < 0.05).

1 Values are expressed as mean ± standard error (*n* = 20 pigs).

2 NBW-C = NBW pigs fed a standard basal diet.

3 IUGR-C = IUGR pigs fed a standard basal diet.

4 IUGR-D = IUGR pigs fed a standard basal diet supplemented with 0.1% DMG-Na.

The results of LM energy metabolites are displayed in Table 7. Compared to the NBW-C group, the IUGR-C group demonstrated a significantly higher (*P* < 0.05) level of NADH and significantly lower (*P* < 0.05) levels of NAD^+^, NAD^+^/NADH ratio, ATP, and mtDNA. Conversely, the IUGR-D group exhibited a lower (*P* < 0.05) NADH level and higher (*P* < 0.05) levels of NAD^+^, NAD^+^/NADH ratio, ATP, and mtDNA compared to the IUGR-C group.

### 3.6 Meat quality assays

The results of LM meat quality are depicted in Table 8. In comparison to the NBW-C group, the IUGR-C group demonstrated significantly higher (*P* < 0.05) levels of drip loss 48 h, L∗, and b∗, and significantly lower (*P* < 0.05) levels of pH 24 h and a∗. Conversely, the IUGR-D group exhibited lower (*P* < 0.05) levels of drip loss 48 h, L∗, and b∗, and higher (*P* < 0.05) levels of pH 24 h and a∗ compared to the IUGR-C group.

**Table 8:** Supplementation with DMG-Na improves the meat quality of LM in IUGR pigs 1.

Item	NBW-C^2^	IUGR-C^3^	IUGR-D^4^
pH 45 min	6.70 ± 0.61	6.64 ± 0.62	6.68 ± 0.66
pH 24 h	5.89 ± 0.55^a^	4.70 ± 0.49^b^	5.82 ± 0.58^a^
Drip loss 24 h, %	9.98 ± 0.88	10.52 ± 0.92	10.16 ± 0.93
Drip loss 48 h, %	15.26 ± 1.48^b^	20.31 ± 1.22^a^	16.13 ± 1.49^b^
Cooking loss, %	27.82 ± 2.52	28.49 ± 2.49	28.11 ± 2.80
L∗	43.25 ± 4.02^b^	52.5 ± 4.56^a^	44.31 ± 4.43^b^
a∗	11.21 ± 1.02^a^	7.07 ± 0.84^b^	11.09 ± 1.09^a^
b∗	17.05 ± 1.55^b^	21.38 ± 1.82^a^	17.22 ± 1.69^b^

DMG-Na = dimethylglycine sodium salt; LM = longissimus dorsi; NBW = normal birth weight; IUGR = intrauterine growth restriction; L∗ = lightness; a∗ = redness; b∗ = yellowness.

^a, b^ Within a row, means with different superscripts indicated significantly different (*P* < 0.05).

1 Values are expressed as mean ± standard error (*n* = 20 pigs).

2 NBW-C = NBW pigs fed a standard basal diet.

3 IUGR-C = IUGR pigs fed a standard basal diet.

4 IUGR-D = IUGR pigs fed a standard basal diet supplemented with 0.1% DMG-Na.

### 3.7 Quantitative reverse transcription PCR assays

**Fig. 2:**
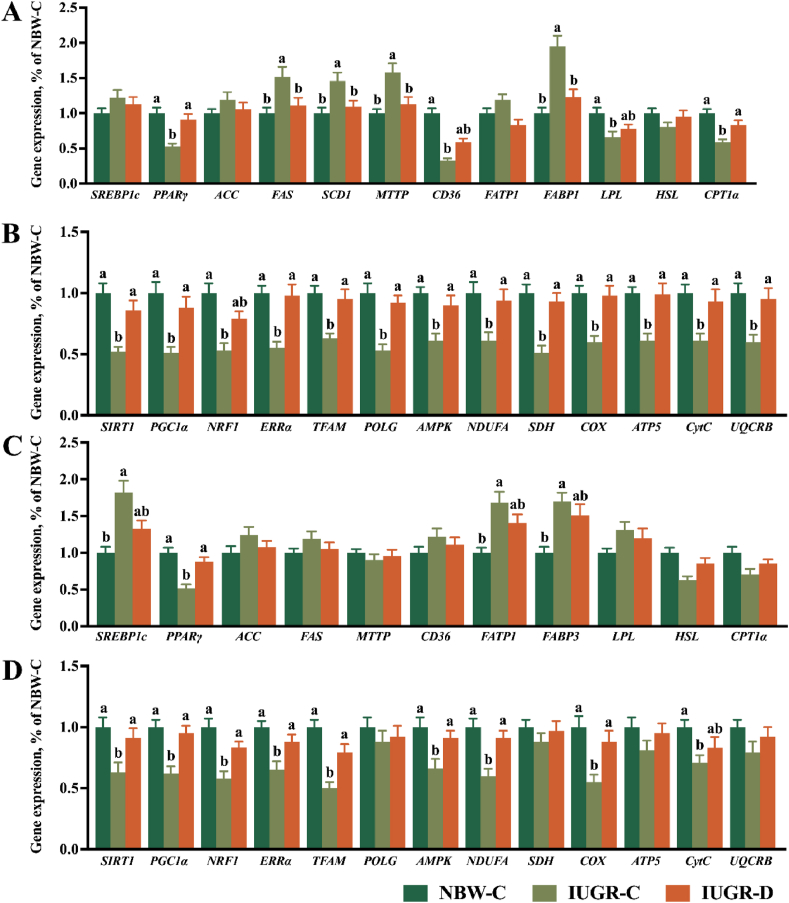
Supplementation with DMG-Na improves the gene expression of liver and LM in IUGR pigs. (A) Lipid metabolism and (B) mitochondrial function gene expression values of the liver, as well as (C) lipid metabolism and (D) mitochondrial function gene expression values of the LM. Data are expressed as the mean ± standard error (*n =* 20 pigs). Different superscripts of a and b represent significant differences (*P* < 0.05). DMG-Na = dimethylglycine sodium salt; LM = longissimus dorsi; NBW = normal birth weight; IUGR = intrauterine growth restriction; *SREBP1c* = sterol regulatory element binding protein 1c; *PPARγ* = peroxisome proliferator activated receptor gamma; *ACC* = acetyl-CoA carboxylase; *FAS* = fatty acid synthase; *MTTP* = microsomal triglyceride transfer protein; *CD36* = cluster of differentiation 36; *FATP1* = fatty acid transport protein 1; *FABP3* = fatty acid binding protein 3; *LPL* = lipoprotein lipase; *HSL* = hormone-sensitive lipase; *CPT1α* = carnitine palmitoyltransferase 1 alpha; *SIRT1* = sirtuin l; *PGC1α* = peroxisome proliferation activated receptor γ coactivator 1α; *NRF1* = nuclear respiratory factor l; *ERRα* = estrogen-related receptor α; *TFAM* = mitochondrial transcription factor A; *POLG* = polymerase; *AMPK* = Adenosine 5′-monophosphate-activated protein kinase; *NDUFA* = NADH dehydrogenase (ubiquinone) 1α subcomplex; *SDH* = succinate dehydrogenase complex flavoprotein subunit; *COX* = cytochrome *c* oxidase; *ATP5* = adenosine triphosphate5; *CytC* = cytochrome *c*; *UQCRB* = ubiquinol-cytochrome c reductase binding protein. NBW-C = normal birth weight pigs fed a standard basal diet; IUGR-C = intrauterine growth restriction pigs fed a standard basal diet; IUGR-D = intrauterine growth restriction pigs fed a standard basal diet supplemented with 0.1% DMG-Na.

The results of hepatic qPCR are depicted in Fig. 2A and B. In comparison to the NBW-C group, the IUGR-C group exhibited significantly higher (*P* < 0.05) levels of *FAS*, *SCD1*, *MTTP*, and *FABP1*, and significantly lower (*P* < 0.05) levels of *PPARγ*, *CD36*, *LPL*, *CPT1α*, *SIRT1*, *PGC1α*, *NRF1*, *ERRα*, *TFAM*, *POLG*, *AMPK*, *NDUFA*, *SDH*, *COX*, *ATP5*, *CytC*, and *UQCRB*. Conversely, the IUGR-D group demonstrated lower (*P* < 0.05) levels of *FAS*, *SCD1*, *MTTP*, and *FABP1*, and higher (*P* < 0.05) levels of *PPARγ*, *CPT1α*, *SIRT1*, *PGC1α*, *ERRα*, *TFAM*, *POLG*, *AMPK*, *NDUFA*, *SDH*, *COX*, *ATP5*, *CytC*, and *UQCRB* compared to the IUGR-C group.

The results of LM qPCR are illustrated in Fig. 2C and D. Compared with the NBW-C group, the IUGR-C group showed significantly higher (*P* < 0.05) levels of *SREBP1C*, *FATP1*, and *FABP3*, and significantly lower (*P* < 0.05) levels of *PPARγ*, *SIRT1*, *PGC1α*, *NRF1*, *ERRα*, *TFAM*, *AMPK*, *NDUFA*, *COX*, *ATP5*, and *CytC*. In contrast, the IUGR-D group exhibited higher (*P* < 0.05) levels of *PPARγ*, *SIRT1*, *PGC1α*, *ERRα*, *TFAM*, *AMPK*, *NDUFA*, and *COX* compared to the IUGR-C group.

### 3.8 Western blotting assays

**Fig. 3:**
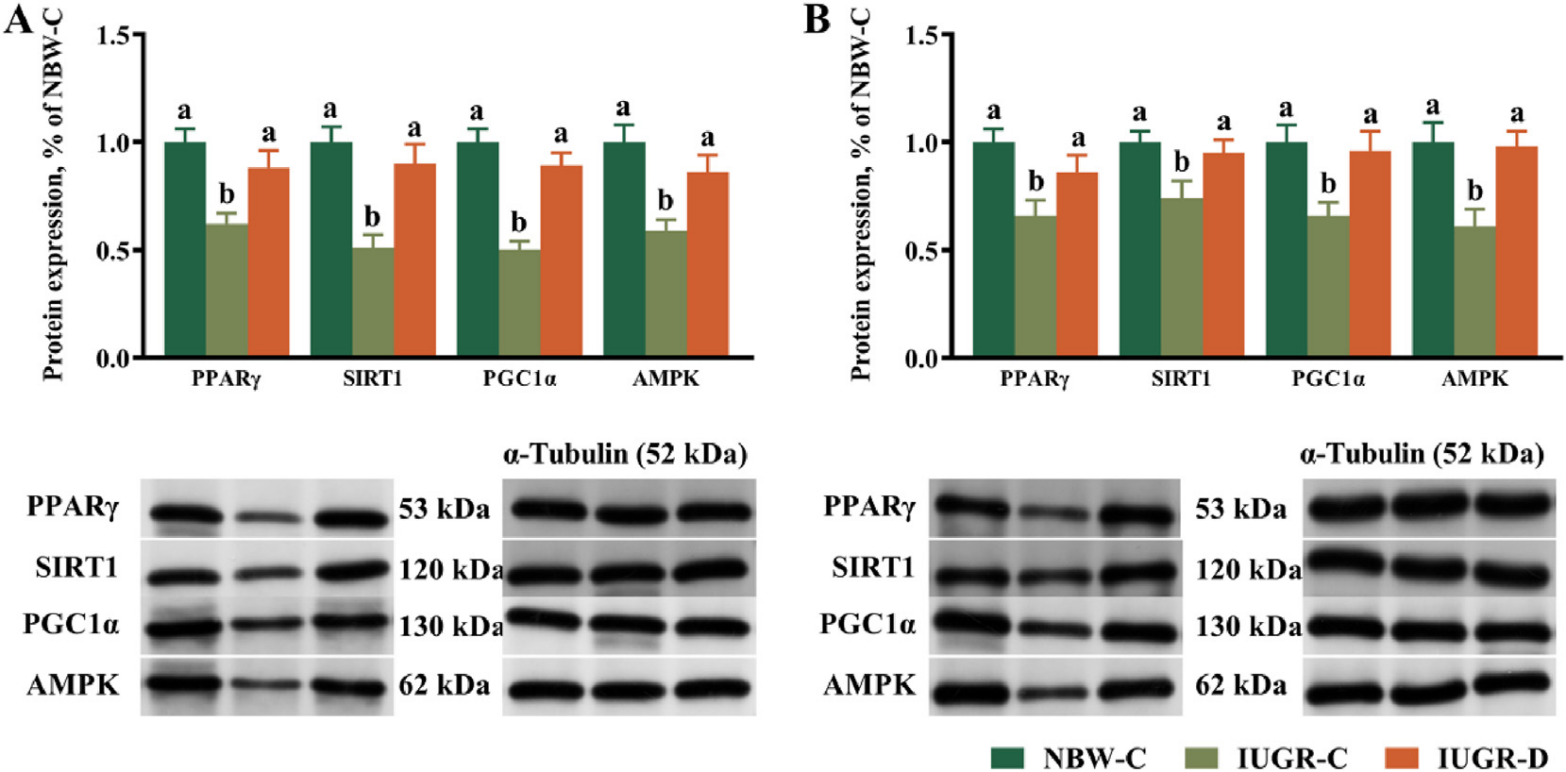
Supplementation with DMG-Na improves the protein expression of liver and LM in IUGR pigs. (A) protein expression values of the liver and (B) protein expression values of the LM. Data are expressed as the mean ± standard error. Different superscripts of a and b represent significant differences (*P* < 0.05). DMG-Na = dimethylglycine sodium salt; LM = longissimus dorsi; NBW = normal birth weight; IUGR = intrauterine growth restriction; PPARγ = peroxisome proliferator-activated receptor gamma; Sirt1 = sirtuin 1; PGC1α = peroxisome proliferator-activated receptor γ coactivator 1α; AMPK = Adenosine 5′-monophosphate (AMP)-activated protein kinase. NBW-C = normal birth weight pigs fed a standard basal diet; IUGR-C = intrauterine growth restriction pigs fed a standard basal diet; IUGR-D = intrauterine growth restriction pigs fed a standard basal diet supplemented with 0.1% DMG-Na.

The results of hepatic western blotting are depicted in Fig. 3A. In comparison to the NBW-C group, the IUGR-C group exhibited significantly lower (*P* < 0.05) levels of PPARγ, SIRT1, PGC1α, and AMPK. Conversely, the IUGR-D group demonstrated higher (*P* < 0.05) levels of PPARγ, SIRT1, PGC1α, and AMPK compared to the IUGR-C group.

The results of LM western blotting are illustrated in Fig. 3B. Compared with the NBW-C group, the IUGR-C group showed significantly lower (*P* < 0.05) levels of PPARγ, SIRT1, PGC1α, and AMPK. In contrast, the IUGR-D group exhibited higher (*P* < 0.05) levels of PPARγ, SIRT1, PGC1α, and AMPK compared to the IUGR-C group.

## 4 Discussion

Intrauterine growth retardation is a condition characterized by delayed fetal growth and development in the uterus, often attributed to genetic or environmental factors (Duhig and Myers, 2022). Studies have shown that IUGR increases the risk of metabolic syndrome in offspring due to persistent disruptions in lipid metabolism postnatally (Chen et al., 2022; Zhang et al., 2023). In pig industry, IUGR is prevalent and has detrimental effects on postnatal growth and development. Throughout the study, all pigs remained in good health, with no instances of mortality observed in any of the experimental groups. In addition, it was observed that the FBW and FI of IUGR-C pigs caught up with those of NBW-C pigs, indicating a phenomenon of catch-up growth. However, catch-up growth, while restoring body weight, often leads to excessive fat accumulation and is considered an independent risk factor for metabolic diseases in IUGR offspring. The increased fat accumulation in liver and skeletal muscle observed further underscores the potential metabolic consequences of catch-up growth, including insulin resistance and abnormal lipid deposition in tissues (Cui et al., 2022; Groene et al., 2023). The study also found that impaired fatty acid oxidation, likely due to mitochondrial dysfunction, contributes to persistent lipid metabolism disorders in IUGR. Interestingly, the supplementation of DMG-Na appeared to mitigate excessive lipid deposition and lipid metabolism disorders in liver and LM tissues, potentially by enhancing mitochondrial energy metabolism in peripheral tissues (Bai et al., 2022a,b, d). The growth-promoting effect and reduction in lipid deposition attributed to DMG-Na may be associated with its potent antioxidant properties, which could augment energy metabolism and facilitate efficient nutrient utilization. Furthermore, the anti-stress effects of DMG-Na may aid in alleviating growth impairment induced by stress (Bai et al., 2021, 2022a,b,c,d). However, the exact mechanism underlying this effect requires further investigation.

Blood lipids, including TC, triglycerides, HDL-C, LDL-C, and FFA, play crucial roles in energy metabolism and are reflective of lipid synthesis and catabolism status in the body. Triglycerides serve as a primary form of energy storage, primarily in adipose tissue, and are mobilized into glycerol and fatty acids when energy demands increase, releasing energy through fatty acid oxidation pathways (Scarmeas and Hooshmand, 2023). In this study, the lower triglyceride levels observed in the IUGR-C group compared to NBW-C group suggest a potential association with the catch-up growth phenomenon seen in these pigs. Free fatty acids, derived mainly from adipose tissue lipolysis, serve as essential substrates for cellular energy metabolism and play roles in regulating glucose and lipid metabolism as well as insulin function (Chaves et al., 2022). The increased plasma FFA levels observed in the IUGR-C group compared to NBW-C group may stem from adipose tissue expansion and heightened lipolysis due to compensatory growth postnatally. The altered cholesterol metabolism in IUGR, reflected by reduced LDL-C levels, could be linked to changes in lipid synthesis and transportation. The decrease in LDL-C may indicate impaired cholesterol transport, potentially leading to elevated FFA levels in the liver and subsequent disturbances in lipid metabolism (Maki et al., 2022; Morris et al., 2022). Despite reduced LPL activity in IUGR pigs, lipid deposition in liver and muscle tissues remains elevated, suggesting an adaptive response to prevent excessive lipid accumulation. dimethylglycine sodium salt supplementation appears to mitigate these abnormalities, possibly by enhancing fatty acid uptake and mitochondrial oxidation in liver and LM tissues, thereby improving lipid and glucose metabolism.

Insulin-like growth factor 1 plays a crucial role in regulating cell growth and differentiation, thereby promoting animal growth (Abdellatif et al., 2022). Reduced levels of IGF-1 in cord blood have been linked to restricted fetal growth and development in the womb (Spiroski et al., 2020). Interestingly, the present study found increased plasma IGF-1 concentrations in IUGR-C pigs compared to NBW-C pigs, potentially attributed to the occurrence of catch-up growth, a phenomenon observed in previous studies (Ferreira et al., 2021; Chen et al., 2024). Insulin, a polypeptide hormone secreted by pancreatic islet cells, regulates blood sugar levels by promoting glycogen and fat synthesis. Its secretion is influenced by changes in blood glucose concentration (Rabinovich, 2023). Insulin resistance, reflected by the HOMA-IR index, signifies weakened insulin sensitivity and inhibits insulin-regulated signaling pathways (York, 2023). In this study, IUGR-C pigs exhibited significantly increased plasma insulin levels and HOMA-IR compared to NBW-C pigs, indicating insulin resistance, possibly associated with elevated FFA levels in tissues. Notably, hyperinsulinemia is a common metabolic phenotype observed in offspring of IUGR. dimethylglycine sodium salt supplementation was found to reduce plasma insulin concentration and HOMA-IR in IUGR pigs, likely attributed to the decrease in FFA concentration induced by DMG-Na. Consequently, DMG-Na's enhancement of mitochondrial function and oxidative utilization of fatty acids in IUGR pigs ultimately improved insulin sensitivity. This result may be attributed to the indirect impact of DMG-Na on lipid metabolism, possibly through enhancing liver and LM functions. By improving the functionality of these organs, DMG-Na may facilitate the normal metabolism and breakdown of lipids, thereby contributing to maintaining lipid metabolism homeostasis.

Mitochondria play a central role in cellular metabolism, generating energy in the form of ATP through oxidative phosphorylation (Casanova et al., 2023; Monzel et al., 2023). However, during this process, ROS are inevitably produced. Normally, the antioxidant defense system efficiently removes ROS, but if not, it can lead to mitochondrial damage and disrupt lipid metabolism (Harrington et al., 2023). The liver, rich in mitochondria, is vital for lipid metabolism, synthesis of fatty acids, cholesterol, and bile, as well as lipid transport. Dysregulation in liver and LM lipid metabolism can affect lipid deposition in tissues like adipose tissue. In this study, IUGR-C pigs showed decreased liver ATP and mtDNA content and an altered NAD^+^/NADH ratio compared to NBW-C pigs, indicating impaired mitochondrial function and reduced energy production. Additionally, the activity of mitochondrial respiratory chain complexes II and III was reduced in IUGR-C pig livers, suggesting impaired oxidative phosphorylation and ATP synthesis. Furthermore, down-regulated transcriptional expression of genes involved in mitochondrial biosynthesis, such as *NRF1*, *TFAM*, *ERRα*, and *PGC1α*, may contribute to the weakened ATP synthesis ability in IUGR-C pig livers. Similarly, in the LM, IUGR pigs exhibited impaired mitochondrial function, with reduced activity of complexes I, II, III, and V, and down-regulated expression of corresponding genes involved in mitochondrial biogenesis. This impaired mitochondrial biogenesis likely contributes to the reduced energy metabolism efficiency in IUGR skeletal muscle. Remarkably, DMG-Na supplementation enhanced mitochondrial function in both liver and LM of IUGR pigs. It increased the activity of complex III in the liver and complexes I, III, and V in the LM, ultimately improving mitochondrial oxidative phosphorylation efficiency and ATP and mtDNA generation. Additionally, DMG-Na alleviated the weakened energy metabolism in IUGR pig LM by enhancing the mitochondrial oxidative phosphorylation system. This could be attributed to the potential effect of DMG-Na on mitochondrial function, potentially enhancing the energy production process within the cell. In cases of IUGR, the fetus experiences a deficit in nutrients, leading to compromised mitochondrial function, which disrupts normal lipid metabolism pathways. This disruption results in abnormal lipid accumulation and metabolic irregularities. Mitochondrial dysfunction can impede the mitochondrial respiratory chain, affecting the crucial oxidative phosphorylation process required for lipid metabolism. Additionally, it can escalate oxidative stress levels, heightening the production of reactive oxygen species and causing damage to cell membranes and lipid molecules, thus exacerbating disturbances in lipid metabolism. Prior studies have suggested that supplementation with DMG-Na could potentially serve as an intervention for mitochondrial dysfunction in IUGR cases (Bai et al., 2021, 2022a,b,c,d). Dimethylglycine sodium salt plays a pivotal role in mitochondrial energy metabolism and possesses antioxidant and cytoprotective properties. By enhancing normal electron transfer in the mitochondrial respiratory chain, DMG-Na boosts cellular energy production, thereby supporting the maintenance of lipid metabolism pathways. Furthermore, its antioxidant properties diminish the generation of reactive oxygen species, thus shielding cell membranes and lipid molecules from oxidative harm and slowing down the progression of lipid metabolism disorders. DMG-Na may also directly impact lipid metabolism pathways by promoting fatty acid oxidation and inhibiting fat synthesis, thus restoring lipid metabolism balance and alleviating disturbances. Overall, DMG-Na supplementation holds promise in ameliorating the impact of mitochondrial dysfunction on lipid metabolism and promoting overall metabolic well-being in cases of IUGR.

Skeletal muscle plays a crucial role in glucose metabolism, as it is responsible for a significant portion of post-feeding glucose uptake and utilization under the stimulation of insulin (Liang et al., 2023; Kopecky et al., 2024). Additionally, skeletal muscle serves as a major site for the oxidation of fatty acids, providing energy for muscle growth, development, and function. When insulin resistance occurs in skeletal muscle, it can lead to elevated blood sugar levels, promoting adipose tissue synthesis from excess glucose (Park et al., 2023). Moreover, skeletal muscle insulin resistance can also disrupt fatty acid metabolism, leading to abnormal lipid deposition in adipose tissue (Li et al., 2023). Intrauterine growth retardation negatively impacts postnatal skeletal muscle growth and glucose metabolism in offspring (Bai et al., 2021, 2022b, d). This can have detrimental effects on meat quality, resulting in changes such as high shear forces, low pH, increased drip loss, and altered meat color. The dyspigmentation observed in the LM of IUGR pigs may be attributed to abnormal lipid deposition and mitochondrial dysfunction. With increased intramuscular lipid deposition, mitochondrial fatty acid oxidation and ROS production also increases, exacerbating mitochondrial dysfunction and oxidative stress. Excessive ROS can damage biological macromolecules, compromising muscle structure and function (Bai et al., 2021, 2022b,d). In this study, DMG-Na supplementation reduced the L∗ and b∗ and increased a∗ of the LM in IUGR-C pigs compared to NBW-C pigs. This improvement in meat quality may be attributed to the antioxidant and lipid-lowering properties of DMG-Na. By reducing oxidative stress and lipid accumulation, DMG-Na helps preserve the integrity of cell membranes, thereby reducing drip loss and improving meat quality. This could be associated with the antioxidant properties of DMG-Na observed in our previous findings (Bai et al., 2021, 2022a,b,c,d). These properties may mitigate the stress and disease on LM tissue during growth, consequently indirectly influencing meat quality by preserving the integrity of LM cells and reducing lipid oxidation, which can impact flavor and texture. IUGR can lead to heightened oxidative stress levels, increasing the production of reactive oxygen species and causing damage to cell membranes and lipid molecules, ultimately worsening lipid metabolism disturbances and meat quality. However, DMG-Na offers robust antioxidant properties that effectively counteract lipid oxidation in muscles, thereby slowing down the process of fatty acid oxidation and lipid degradation. This mechanism helps to preserve the tenderness and flavor of the muscle tissue. Notably, unsaturated fatty acids are particularly vulnerable to oxidative damage, which can result in the development of undesirable odors and flavors while compromising muscle quality. By incorporating DMG-Na as a potent antioxidant, the freshness and nutritional integrity of unsaturated fatty acids in the muscle tissue can be effectively maintained.

The regulation of lipid metabolism in animals involves complex interactions between various tissues, cells, and molecules. The liver is responsible for synthesizing, transporting, and oxidizing fatty acids, while skeletal muscle, being highly distributed throughout the body, primarily relies on fatty acids for energy consumption (Cook et al., 2023; Koves et al., 2023). Crosstalk between these tissues is crucial for maintaining lipid metabolism homeostasis. Several proteins and enzymes contribute to lipid metabolism regulation, including LPL, microsomal triglyceride transfer protein (MTTP), fatty acid transport proteins (FATPs), and fatty acid binding proteins (FABPs) (Samovski et al., 2023; Schneider et al., 2023; Yan et al., 2023). Enzymes involved in lipogenesis (e.g., acetyl coenzyme A carboxylase, fatty acid synthase, stearoyl-CoA desaturase) and lipolysis (e.g., hormone-sensitive lipase, adipose triglyceride lipase, carnitine palmitoyltransferase 1) also play crucial roles (Jeon et al., 2023; Yang et al., 2023). Transcription factors such as peroxisome proliferator-activated receptor α (PPARα) and sterol regulatory element-binding protein 1c (SREBP1c) regulate the expression of genes involved in lipid metabolism (Jeong et al., 2023; Zhong et al., 2023). In the context of IUGR, abnormal lipid deposition in the liver may result from increased lipid transport and de novo synthesis, driven by upregulated expression of *SREBP1c*, stearoyl-CoA desaturase 1 (*SCD1*), and fatty acid binding protein 1 (*FABP1*) genes. Interestingly, although DMG-Na supplementation increased LPL activity and gene expression, it still reduced lipid deposition in the liver and LM of IUGR pigs. The mechanism of action of DMG-Na may differ between the liver and LM. In the liver, DMG-Na may improve mitochondrial function, while in LM, it may enhance lipid uptake, transport, breakdown, and oxidation. The role of PPARα in DMG-Na's effects on lipid metabolism cannot be overlooked. Peroxisome proliferation activated receptor γ coactivator 1α (PGC1α), a master regulator of mitochondrial biogenesis, orchestrates the expression of genes involved in oxidative phosphorylation, mitochondrial respiration, and energy metabolism (Ozaki et al., 2023). Activation of sirtuin l (SIRT1) enhances PGC1α and AMPK activity, promoting mitochondrial biogenesis and improving mitochondrial function by stimulating the expression of relative genes, including nuclear respiratory factor l (*NRF1*), estrogen-related receptor α (*ERRα*), mitochondrial transcription factor A (*TFAM*), polymerase (*POLG*), NADH dehydrogenase (ubiquinone) 1α subcomplex (*NDUFA*), succinate dehydrogenase complex flavoprotein subunit (*SDH*), cytochrome *c* oxidase (*COX*), cytochrome *c* (*CytC*) (Deng et al., 2023; Steinberg and Hardie, 2023). This cooperative action leads to increased mitochondrial mass, improved oxidative capacity, and enhanced energy production. Additionally, SIRT1-mediated deacetylation of PGC1α enhances its transcriptional activity, further amplifying the expression of mitochondrial genes (Bai et al., 2022a,b,d). Past research has demonstrated that DMG-Na can improve tissue mitochondrial function (Bai et al., 2021, 2022a,b,c,d). Therefore, DMG-Na likely improves lipid metabolism in IUGR pigs by enhancing mitochondrial function in the liver and skeletal muscle, as well as promoting fatty acid oxidation. However, the current understanding of how DMG-Na alleviates IUGR-induced lipid metabolism disorders in pigs is still limited. Based on our previous studies on IUGR lipid metabolism and the results of this experiment, it appears that DMG-Na indirectly influences lipid metabolism by improving liver and LM functions. Additionally, DMG-Na could enhance energy metabolism, which is closely linked to lipid metabolism's energy production and utilization processes. Hence, based on the interaction between SIRT1/PGC1α and AMPK, this study revealed that DMG-Na activates SIRT1 and AMPK, thereby enhancing the activity of PGC1α and promoting mitochondrial biogenesis and function. Additionally, activation of this pathway by DMG-Na not only regulates lipid metabolism but may also influence multiple cellular physiological processes such as oxidative stress, energy metabolism, and inflammatory responses. Current and future research directions should prioritize investigating the specific regulatory mechanisms of DMG-Na on the activation of the SIRT1/PGC1α-AMPK pathway. This will facilitate the identification of new targets and strategies for the comprehensive treatment of related diseases.

The study provides valuable insights into the effects of DMG-Na on mitochondrial function and lipid metabolism through the SIRT1/PGC1α-AMPK pathway. However, several limitations should be acknowledged. Firstly, the study exclusively involved male pigs, potentially overlooking gender-specific metabolic responses. Additionally, focusing on a single pig breed may limit the generalizability of the findings to other breeds, which could exhibit different metabolic responses. Moreover, the controlled laboratory conditions may not fully capture the complexity and variability of real-world pig production settings, where factors such as environmental conditions, feeding practices, and genetic diversity can significantly influence metabolic processes. Therefore, further research is needed to address these limitations and enhance the applicability of the findings.

## 5 Conclusions

In conclusion, the current study revealed that DMG-Na effectively ameliorated lipid metabolism disorder in IUGR pigs. We found that DMG-Na directly enhanced hepatic and LM mitochondrial function, indirectly improved lipid metabolic abnormalities, and inhibited the abnormal expression of lipid metabolism and mitochondrial function-related factors. These findings suggest that DMG-Na may serve as a health-promoting substance for the prevention of lipid metabolism disorders.

## Author contributions

**Kaiwen Bai:** Conceptualization, Methodology, Validation, Formal analysis, Investigation, Writing – original draft, Funding acquisition. **Luyi Jiang:** Investigation, Writing – review & editing, Supervision. **Tian Wang:** Writing – review & editing, Supervision.

## Declaration of competing interest

We declare that we have no financial and personal relationships with other people or organizations that can inappropriately influence our work, and there is no professional or other personal interest of any nature or kind in any product, service and/or company that could be construed as influencing the content of this paper.
