# Supplementary material for: Supplementation with dimethylglycine sodium salt improves lipid metabolism disorder in intrauterine growth-retarded pigs
Source: Anim Nutr. 2024 Jun 1;18:191–202. doi: 10.1016/j.aninu.2024.05.002 (PMC11393594; doi:10.1016/j.aninu.2024.05.002)
Supplement: Multimedia component 1 [file mmc1.docx]

**Table S1. Composition and nutrient levels of the diets for sows^1^ (as-fed basis, %).**

| Ingredients | Content | Ingredients | Content |
| --- | --- | --- | --- |
| Ingredients |  | Analyzed nutrient concentrations |  |
| Corn | 54.00 | CP | 15.60 |
| Soybean meal | 18.00 | CF | 3.2 |
| Wheat bran | 10.00 | Lysine | 1.01 |
| Wheat middling | 10.00 | Ca | 0.78 |
| Fish meal | 1.50 | P | 0.54 |
| Soybean oil | 2.50 | Calculated nutrient level |  |
| Premix^1^ | 4.00 | DE, MJ/kg | 13.46 |
| Total | 100.00 | ME, MJ/kg | 12.80 |

^1^Provided per kilogram of diet: vitamin A, 8,000 IU; vitamin D_3_, 2,000 IU; vitamin E, 40 mg; vitamin K, 2 mg; vitamin B_1_, 3 mg; vitamin B_2_, 5 mg; vitamin B_6_, 3 mg; vitamin B_12_, 0.04 mg; nicotinic acid, 30 mg; pantothenic acid, 20 mg; folic acid, 1.5 mg; biotin, 0.3 mg; Fe, 100 mg; Cu, 20 mg; Zn, 80 mg; Mn, 40 mg; I, 0.3 mg; Se, 0.25 mg.

**Table S2. Primer sequences used for Real-time PCR assay.**

| Gene^1^ | Primer sequence (5’ → 3’) | Genbank ID^2^ | Gene^1^ | Primer sequence (5’ → 3’) | Genbank ID^2^ |
| --- | --- | --- | --- | --- | --- |
| β-Actin | GTTCGAGACCTTCAACACGC | XM_003357928.3 | *PGC1α* | GGGGCCCATGGGAATCATC | XM_013992150.1 |
|  | CCATGACAATGCCAGTGGTG |  |  | AACTGCTGTTGTTTGGGCCT |  |
| *SREBP1c* | GCGACGGTGCCTCTGGTAGT | NM_214157.1 | *NRF1* | TTGCAGAGGTGCAATCAAATGG | XM_013985624.1 |
|  | CGCAAGACGGCGGATTTA |  |  | TCCAAAATCCGCTGCCTTTC |  |
| *PPARγ* | GGCACTGAACATCGAATGTAGAAT | NM_001044526.1 | *ERRα* | GGTGGGCGACAGAAGTACAA | NM_001170521.1 |
|  | TGCAACCTTCACAGGCATGA |  |  | ACCACAATCTCTCGGTCGAA |  |
| *ACC* | ATCCCTCCTTGCCTCTCCTA | NM_001114269.1 | *TFAM* | TATAGGGCAGACTGGCAGGT | NM_001130211.1 |
|  | ACTTCCCGTTCAGATTTCCG |  |  | TGGACCATCCTTAGCTTCCT |  |
| *FAS* | TACCTTGTGGATCACTGCATAGA | NM_001099930.1 | *POLG* | AGCAGAAGCCCCAAAGTTCC | XM_001927064.4 |
|  | GGCGTCTCCTCCAAGTTCTG |  |  | AGCATGACCTCTCTCCCTTCT |  |
| *MTTP* | AGCAAAATGGTCCGTCGAGT | NM_214185.1 | *AMPK* | AAATCGGCCACTACATCCTG | NM_001167633.1 |
|  | CGAATGGGGACCACGTTCTA |  |  | GGATGCCTGAAAAGCTTGAG |  |
| *CD36* | TGACCCAGCACTTGAAGCAA | NM_001044622.1 | *NDUFA* | TGCTAAGTGGCAAAGCCTGA | XM_003124046.3 |
|  | AAGATATCAGTTAGGAGTCCGATGA |  |  | GAACAAAACATCCGGAGGCG |  |
| *FATP1* | AGGTCTGGCGTGGGTCAAAG | NM_001083931.1 | *SDH* | ATGGAAAACGGGGAGTGTCG | CX063991.1 |
|  | GGAGTAGAGGGCAAAGCAGG |  |  | TTCCGGTAGCGACAACAGTG |  |
| *FABP3* | TTGTGACACTGGATGGAGGC | NM_001099931.1 | *COX* | GCAGTTGCCAGATGCTGAAC | XM_001926129.5 |
|  | TAAGTGCGAGTGCAAACTGC |  |  | TGGGTGAAGTGTTGGGCAAA |  |
| *LPL* | CACATTCACCAGAGGGTC | NM_214286.1 | *ATP5* | ACGCCATTGATGGAAAGGGT | NM_001185142.1 |
|  | TCATGGGAGCACTTCACG |  |  | TGGTTCCCGCACAGAGATTC |  |
| *HSL* | GCAGCATCTTCTTCCGCACA | NM_214315.3 | *CytC* | TCGGAGTCACCAGTGCTAGA | XM_003127002.3 |
|  | AGCCCTTGCGTAGAGTGACA |  |  | GGAACATTGAGGCCTACGGA |  |
| *CPT1α* | TCAAAAACGGCAAGATGGGC | NM_001129805.1 | *UQCRB* | CATCAGGCAACGCTTCTGTC | NM001185172.1 |
|  | TGGAATGTTGGGGTTGGTGT |  |  | TATACCCTCCAGCCACTTGC |  |
| *SIRT1* | GGTGGTTCCTCGATGTCCTA | NM_001145750.1 |  |  |  |
|  | GGTGAGGCAAAGGTTCCCTA |  |  |  |  |

^1^ *SREBP1c* = sterol regulatory element binding protein 1c; *PPARγ* = peroxisome proliferator activated receptor gamma; *ACC* = acetyl-CoA carboxylase; *FAS* = fatty acid synthase; *MTTP* = microsomal triglyceride transfer protein; *CD36* = cluster of differentiation 36; *FATP1* = fatty acid transport protein 1; *FABP3* = fatty acid binding protein 3; *LPL* = lipoprotein lipase; *HSL* = hormone-sensitive lipase; *CPT1α* = carnitine palmitoyltransferase 1 alpha; *SIRT1* = sirtuin l; *PGC1α* = peroxisome proliferation activated receptor γ coactivator 1α; *NRF1* = nuclear respiratory factor l; *ERRα* = estrogen-related receptor α; *TFAM* = mitochondrial transcription factor A; *POLG* = Polymerase; *AMPK* = Adenosine 5’-monophosphate-activated protein kinase; *NDUFA* = NADH dehydrogenase (ubiquinone) 1α subcomplex; *SDH* = succinate dehydrogenase complex flavoprotein subunit; *COX* = cytochrome c oxidase; *ATP5* = adenosine triphosphate5; *CytC* = cytochrome c; *UQCRB* = ubiquinol-cytochrome c reductase binding protein.

^2^ GenBank Accession Number.
